# Severe immunotherapy-related autoimmune hemolytic anemia induced by toripalimab in a patient with deficient mismatch repair colorectal cancer: a case report and literature review

**DOI:** 10.3389/fmed.2025.1678754

**Published:** 2025-10-13

**Authors:** Xu Ma, Jin-Sheng Ye, Zhi Liu, Quan-Li Geng

**Affiliations:** ^1^Department of General Surgery, Beijing Yanqing Hospital of Traditional Chinese Medicine, Beijing, China; ^2^Department of General Surgery/Oncology, Beijing Hospital of Traditional Chinese Medicine, Capital Medical University, Beijing, China

**Keywords:** immune checkpoint inhibitors, toripalimab, dMMR colorectal cancer, immunotherapy-related autoimmune hemolytic anemia, immune-related adverse event

## Abstract

Immune checkpoint inhibitors (ICIs) have recently emerged as a promising class of anticancer therapy, demonstrating significant efficacy across various malignancies. They are currently regarded as the first-line therapy for advanced mismatch repair-deficiency colorectal cancer. However, the extensive clinical usage of ICIs has raised concerns regarding immune-related adverse events (irAEs). Herein, we describe a case of immunotherapy-related autoimmune hemolytic anemia (irAIHA) in a patient with locally advanced mismatch repair-deficiency colorectal cancer treated with toripalimab, a programmed cell death 1 (PD-1) (ICI). The patient developed grade 4 irAIHA after the first cycle of immunotherapy, which was promptly managed by discontinuing treatment and initiating high-dose prednisone. Symptoms were controlled, and hemoglobin returned to normal without resuming immunotherapy. Although hematologic irAEs such as irAIHA are relatively rare, they can be life-threatening and require immediate intervention. This case underscores the importance of vigilant monitoring, early recognition, and timely, aggressive management of irAEs during ICI therapy. In high-risk populations, including elderly patients with comorbidities, the toxicities associated with corticosteroid therapy pose additional challenges, emphasizing the need for individualized strategies that balance efficacy and safety.

## 1 Introduction

Mismatch repair-deficiency (dMMR) colorectal cancer is specifically delineated in the 2023 National Comprehensive Cancer Network Guidelines, which recommend immune checkpoint inhibitors (ICIs) as first-line (1) therapy. Specifically, programmed cell death protein 1/programmed death-ligand 1 (PD-1/PD-L1) inhibitors (e.g., toripalimab) are recommended for patients with advanced or metastatic dMMR colorectal cancer (2). Toripalimab, the first PD-1 inhibitor approved by the National Medical Products Administration of China, is now widely used to treat various solid tumors (3). However, its widespread application may be accompanied by immune-related adverse events (irAEs) that pose significant concerns related to patient safety. Among these, immunotherapy-related autoimmune hemolytic anemia (irAIHA), although rare, is particularly severe and requires immediate medical intervention (4). Its underlying mechanism involves autoimmune activation, wherein the immune system erroneously targets and destroys red blood cells (5). Clinicians should remain highly vigilant in terms of recognizing and managing irAEs, particularly severe irAEs such as irAIHA. Given the severity of irAEs, early recognition and aggressive management are crucial. Herein, we report a case of severe irAIHA in a patient who was receiving toripalimab for dMMR colorectal cancer, highlighting the importance of clinical vigilance and prompt intervention to reduce the risk of life-threatening complications.

## 2 Case presentation

**Figure 1:**
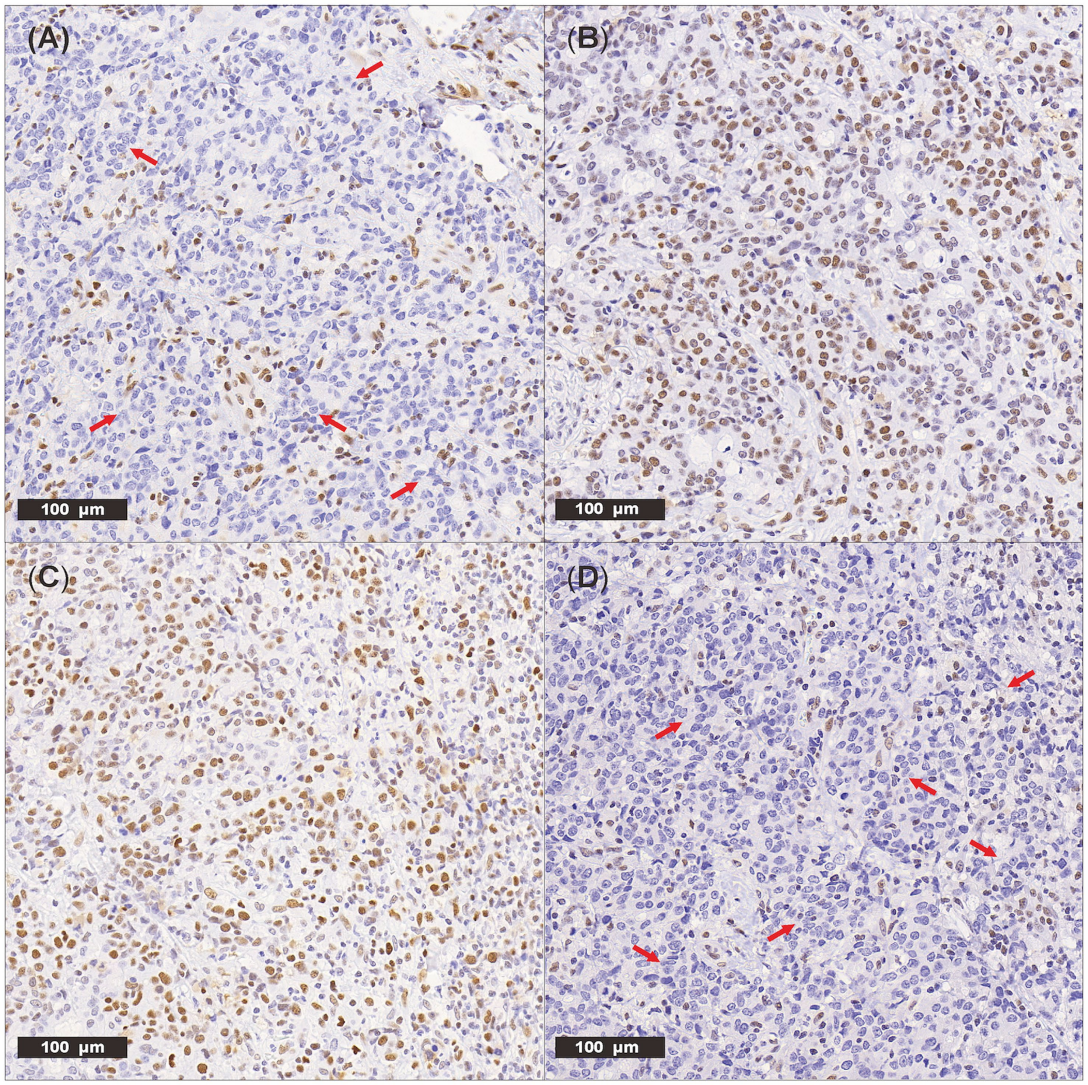
**(A)** Loss of nuclear expression of MLH1(red arrows) by IHC, 20.0× magnification. **(B)** Intact nuclear expression of MSH2 by IHC, 20.0× magnification. **(C)** Intact nuclear expression of MSH6 by IHC, 20.0× magnification. **(D)** Loss of nuclear expression of PMS2(red arrows) by IHC, 20.0× magnification.

An 82-year-old woman, with a body weight of 47.5 kg and a medical history of hypertension and type 2 diabetes, presented with abdominal pain and was diagnosed with a malignant colonic mass. Preoperative laboratory tests performed on February 25, 2023, revealed moderate anemia with a hemoglobin (Hb) level of 89 g/L and reticulocyte percentage (Ret%) of 1.2%, with the latter indicating an inadequate compensatory bone marrow response. Test results also revealed a lactate dehydrogenase (LDH) level of 182 U/L, total bilirubin (TBIL) level of 10.12 μmol/L, and indirect bilirubin (IBIL) level of 5.82 μmol/L. As these parameters were within normal limits, chronic non-hemolytic anemia was suspected. This baseline level of anemia was consistent with the patient’s age and underlying malignancy. The patient underwent a right hemicolectomy on February 26, 2023. Postoperative pathological analysis confirmed a medullary carcinoma measuring 6 × 5 × 5.5 cm, with neural invasion and vascular tumor thrombi, classified as stage pT4N2aM0. Immunohistochemistry confirmed it to be dMMR (Figure 1).

On April 5, 2023 (approximately 1 month postoperatively), the patient began receiving toripalimab (142 mg administered every 14 days). Pre-treatment laboratory tests performed on April 2, 2023, showed a Hb level of 82 g/L, Ret% of 1.4%, LDH of 211 U/L, TBIL of 13.16 μmol/L, and IBIL of 8.16 μmol/L. On April 21, 2023, the patient developed fatigue and jaundice prior to her second scheduled dose of toripalimab. Repeat laboratory tests revealed a drop in the Hb level to 45 g/L and an increase in the Ret% to 9. Her LDH level was also elevated to 856 U/L, and her bilirubin levels were increased (TBIL, 187.35 μmol/L; IBIL, 141.38 μmol/L). The direct antiglobulin test (Coombs test) was positive. Given the close temporal association between symptom onset and toripalimab initiation, the classic biochemical features of hemolysis (sharp hemoglobin decline, elevated LDH to 856 U/L and IBIL to 141.38 μmol/L), and the positive direct antiglobulin test, a clinical diagnosis of Common Terminology Criteria for Adverse Events (CTCAE) grade 4 irAIHA was made (6). Other causes of hemolysis, such as microangiopathic anemia or active bleeding, were ruled out based on a comprehensive clinical assessment (see Table 1).

**Table 1:** Timeline of clinical management and laboratory monitoring.

Indicator/management	Reference range	2023-02-25	2023-04-02	2023-04-21	2023-04-25	2023-04-26	2023-05-05	2023-05-25	2023-07-25
Clinical Event	–	Pre–op Assessment	Pre–IT	Post–IT G4 irAIHA	Transfusion support	Steroid side effects	Condition stable	Condition stable	Condition stable
Key Management	–	–	–	Hold IT Start steroids	Transfuse 2U PRBCs	Steroid taper Cardiac & Glu Monitor	Steroid taper	Steroid taper	D/C Steroids D/C IT permanently
Prednisone Dose	–	–	–	50 mg/day	50 mg/day	30 mg/day	20 mg/day	10 mg/day	Discontinued
Hb (g/L)	110–150	89	82	45	–	57	76	82	87
Ret (%)	0.5–1.5	1.2	1.4	9.0	–	5.0	3.4	1.7	1.8
LDH (U/L)	109–245	182	211	856	–	812	612	487	255
TBIL (μmol/L)	3.42–20.52	10.12	13.16	187.35	–	43.35	–	–	10.16
IBIL (μmol/L)	0–15	5.82	8.16	141.38	–	24.38	–	–	4.50
pro-BNP (pg./mL)	0–125	415.80	242.90	5418.00	–	10,007.00	6812.00	4861.00	397.20
Direct antiglobulin test	–	–	–	Positive	–	–	–	Negative	–

D/C, discontinued; Glu, glucose; Hb, hemoglobin; IBIL, indirect bilirubin; irAIHA, immunotherapy-related autoimmune hemolytic anemia; IT, immunotherapy; LDH, lactate dehydrogenase; PRBCs, packed red blood cells; pro-BNP, pro-hormone of brain natriuretic peptide; Ret, reticulocyte; Steroid, corticosteroid; TBIL, total bilirubin; U, units.

Following the diagnosis of grade 4 irAIHA on April 21, 2023, after toripalimab had been administered, immunotherapy was immediately discontinued in favor of oral prednisone at an initial dose of 50 mg daily. The patient received a transfusion of two units of type B-positive packed red blood cells on April 25, 2023. Follow-up on April 26, 2023, showed that the patient’s Hb level had risen to 57 g/L with a Ret% of 5%, indicating initial control of the hemolytic anemia. However, the high-dose corticosteroid therapy also induced significant adverse effects: the patient’s pro-B-type natriuretic peptide level increased significantly to 10,007 pg./mL, indicating increased cardiac load. The patient’s blood glucose level also became poorly controlled, with fasting levels peaking at 23 mmol/L. To balance the treatment efficacy with drug toxicity, the prednisone dose was reduced to 30 mg daily on April 26, 2023, alongside intensified monitoring of cardiac function and glycemic control. Subsequent assessments confirmed stable Hb levels, and the patient’s cardiac condition was managed using supportive treatment. Following an increase in the patient’s Hb level to 76 g/L on May 5, 2023, the prednisone dose was cautiously reduced further to 20 mg daily. Notably, the patient did not receive any other adjunctive therapies aimed at correcting her anemia, such as intravenous iron supplementation or erythropoiesis-stimulating agents, during the treatment course.

**Figure 2:**
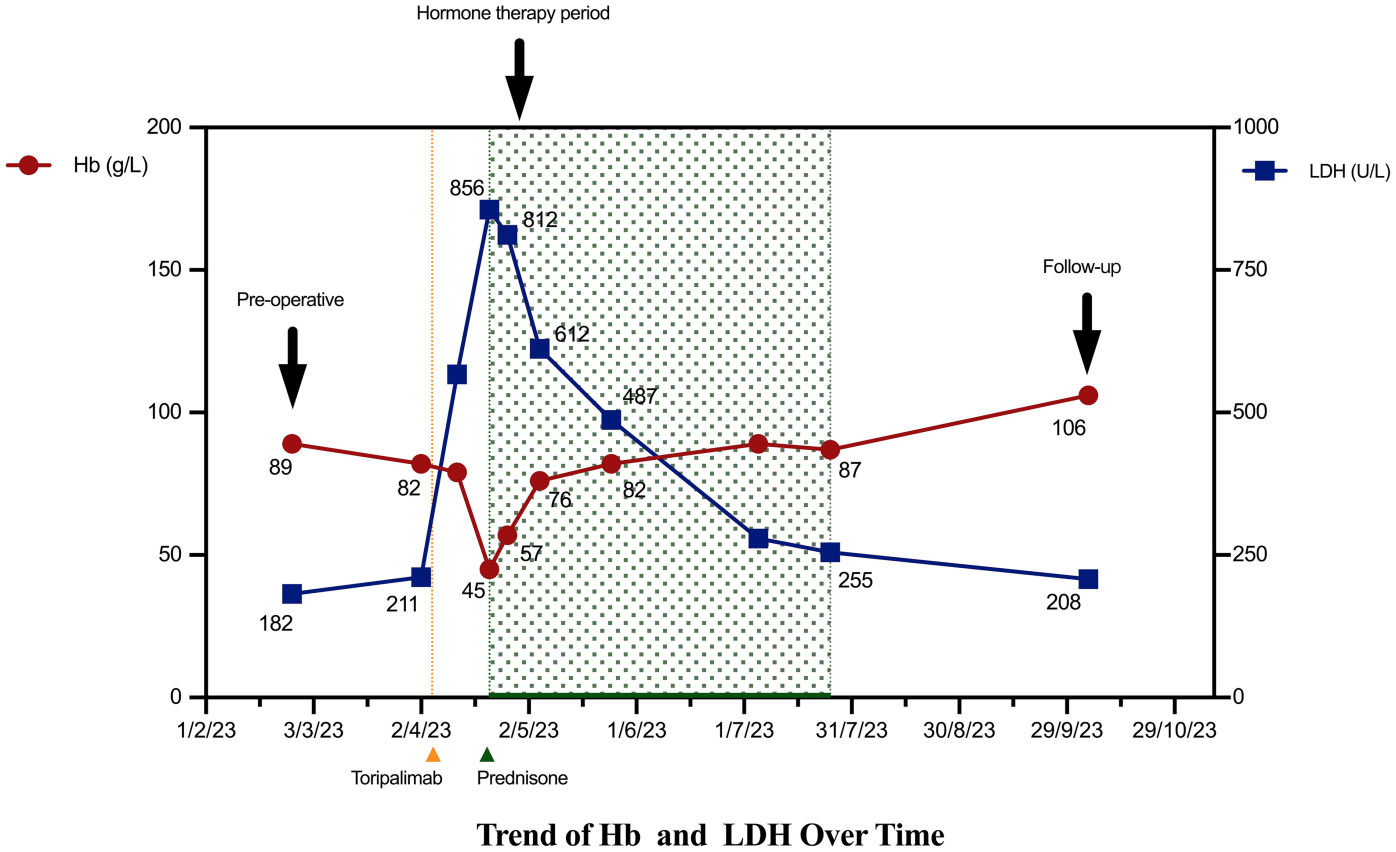
The patient’s hemoglobin (Hb) and lactate dehydrogenase (LDH) levels over time.

During subsequent outpatient follow-ups on May 25 and July 25, 2023, the patient’s hematologic parameters continued to normalize and remained stable. A follow-up direct antiglobulin test showed negative results, demonstrating sustained recovery from the hemolytic episode without recurrence. Given the severity of this adverse event, immunotherapy was permanently discontinued (Figure 2). The reporting of this case conforms to the CARE guidelines (Supplementary file).

## 3 Discussion

PD-1 blockade is recommended by current clinical guidelines (7) as first-line strategy for dMMR metastatic colorectal cancer. Although PD-1 inhibitors provide significant survival benefits and may even cure this cancer, their clinical application is often accompanied by irAEs caused by off-target immune activity, which merit careful attention. The incidence of fatal irAEs is estimated to be between 0.3 and 1.3%, with these events typically occurring early during treatment (8). According to the 2024 National Comprehensive Cancer Network guidelines, ICIs should be immediately discontinued if severe irAEs (i.e., grade 3–4) are detected. High-dose corticosteroid therapy should then be initiated (9), with tapering to lower doses over 4–6 weeks recommended (10). Current guidelines explicitly recommend considering permanent discontinuation of immunotherapy for treating the primary malignancy whenever irAEs with CTCAE grades ≥ 4 are present (11). Therefore, early recognition of and timely intervention for irAEs are crucial to reduce the risk of treatment interruption, maintain quality of life in affected patients, and minimize patients’ mortality risks (12).

Hematologic irAEs are relatively rare but often life-threatening. This report describes rare and life-threatening hematologic toxicity (irAIHA) in an older patient with locally advanced dMMR colorectal cancer after toripalimab administration. Available data indicate that the incidence of irAIHA in patients receiving ICI therapy is approximately 0.6% (10), with reported mortality rates up to 15% (13). This underscores both the exceptional rarity and critical severity of the present case.

The diagnosis of toripalimab-induced CTCAE grade 4 irAIHA was supported by the clear temporal relationship to drug administration, the absence of a prior autoimmune disease history, and complete resolution of symptoms upon treatment withdrawal. Given this life-threatening grade 4 irAIHA, combined with the patient’s age and complex comorbidities, we strictly adhered to current guideline recommendations (14) by immediately and permanently discontinuing toripalimab treatment without switching to another ICI. Although high-dose corticosteroid therapy was rapidly effective, the associated toxicities (e.g., increased cardiac load with a sharp increase in the pro-B-type natriuretic peptide level and loss of glycemic control) necessitated an accelerated steroid taper. While necessary, this may have prolonged the time to complete hematologic recovery. The patient exhibited a favorable and sustained response to first-line corticosteroid treatment, with Hb levels steadily recovering, thereby obviating the need for second-line interventions such as rituximab or intravenous immunoglobulin treatment (15).

This case shares similarities with early-onset, severe AIHA induced by pembrolizumab, as reported by Adeoye et al. (16) and Back et al. (17), supporting the possibility that such hyperacute hematologic toxicity may represent a class effect of PD-1 inhibitors. However, in contrast to the fatal steroid-refractory case reported by Palla et al. (18), our patient responded well to corticosteroid therapy. Although recent case reports have described successful rechallenge with another PD-1 inhibitor (e.g., switching from pembrolizumab to dostarlimab) (19), the extreme severity of the grade 4 toxicity in this case, coupled with the patient’s age and multiple comorbidities, led us to a different clinical decision—namely, not to reinstitute any form of immunotherapy. This underscores the particular challenges of managing irAEs in such high-risk populations; the core of therapeutic decision-making lies in balancing the efficacy of immunosuppression against its inherent risks.

Management of irAIHA differs fundamentally from that of conventional AIHA. Management of AIHA focuses on controlling hemolysis by diagnosing and treating the underlying cause (e.g., lymphoma or infection) (20), whereas the pivotal decision in irAIHA is immediate discontinuation of the offending immune checkpoint inhibitor (21). The treatment goal is to control life-threatening toxicity while balancing the patient’s need for subsequent anticancer therapy. This strategic difference implies that future clinical efforts should shift earlier in the care pathway: high vigilance, early recognition, and rapid intervention are paramount. Accordingly, we advocate for institutional irAE emergency-response protocols and guidelines that provide more detailed strategies to optimize the management of glucocorticoid-induced toxicities and plan post-discontinuation therapy. This integrated approach is essential to safeguard patient safety and long-term quality of life without compromising treatment efficacy.

## Data Availability

The original contributions presented in the study are included in the article/Supplementary material, further inquiries can be directed to the corresponding author.
